# A Survey of Latest Wi-Fi Assisted Indoor Positioning on Different Principles

**DOI:** 10.3390/s23187961

**Published:** 2023-09-18

**Authors:** Jihan Dai, Maoyi Wang, Bochun Wu, Jiajie Shen, Xin Wang

**Affiliations:** 1 School of Computer Science, Fudan University, Shanghai 200438, China; 21210240003@m.fudan.edu.cn (J.D.); wangmaoyi@fudan.edu.cn (M.W.); xinw@fudan.edu.cn (X.W.); 2 Informatization Office, Fudan University, Shanghai 200433, China; wubochun@fudan.edu.cn

**Keywords:** Wi-Fi, indoor positioning, angle-of-arrival, received signal strength indication, time-of-arrival

## Abstract

As the location-based service (LBS) plays an increasingly important role in real life, the topic of positioning attracts more and more attention. Under different environments and principles, researchers have proposed a series of positioning schemes and implemented many positioning systems. With widely deployed networks and massive devices, wireless fidelity (Wi-Fi) technology is promising in the field of indoor positioning. In this paper, we survey the authoritative or latest positioning schemes for Wi-Fi-assisted indoor positioning. To this end, we describe the problem and corresponding applications, as well as an overview of the alternative methods. Then, we classify and analyze Wi-Fi-assisted indoor positioning schemes in detail, as well as review related work. Furthermore, we point out open challenges and forecast promising directions for future work.

## 1. Introduction

With the development of technology, the location information of people and objects can be obtained with ease. Location-based service (LBS) changes the way people live and plays an increasingly important role in various fields, e.g., navigation, logistics, outdoor games, tracking of key personnel, disaster relief, and advertisement. Researchers and engineers have developed various positioning schemes to support the LBS.

Wireless positioning can be classified into outdoor and indoor scenarios. While outdoor positioning technologies have been maturely developed and widely adopted in civilian and military fields [1,2], they are still barely satisfactory in indoor scenarios for two reasons. First, the complex indoor environment complicates the signal propagation [3]. Second, the occlusive adjoining wall reduces the signal strength of the positioning system [4]. Thus, the accuracy of the positioning system suffers in the indoor environment. Instead, compared with the outdoor scenario, the indoor scenario requires higher positioning accuracy for LBS. Moreover, people may usually spend much more time indoors than outdoors [5], which enlarges the demand for indoor positioning.

The technology of wireless fidelity (Wi-Fi) has been attracting extensive attention from researchers due to its wide coverage, high popularity, and low power consumption. As Wi-Fi protocol standards are continually upgraded, corresponding networks and devices are rapidly iterating. The latest Wi-Fi 7 newly releases 6 GHz frequency band with a greater channel bandwidth. An increasing number of Wi-Fi devices are equipped with fine timing measurement (FTM) [6] and channel state information (CSI) measurement. These are beneficial for improving positioning accuracy. Therefore, Wi-Fi is a promising solution to indoor positioning.

To the best of our knowledge, this paper is a brand-new survey to comprehensively review state-of-the-art work of Wi-Fi-assisted indoor positioning in recent years. The review [7] also provides a systematic summary of Wi-Fi-assisted indoor positioning. However, compared to this, our review has distinct classification standards for Wi-Fi-assisted indoor positioning, and the references involved in our review are more comprehensive and updated.

The main contributions of this paper are summarized as follows:

- Basic description and analysis of indoor positioning. This paper describes the indoor positioning problem and its applications. We state the key issues in anti-interference and practical deployment. We also outline mainstream alternative methods and compare their advantages and disadvantages.
- Classification and review of related work. According to the positioning principle, we divide Wi-Fi-assisted indoor positioning schemes into three categories. We state the principles of these categories and point out their merits and demerits. We also review representative work of corresponding simple and hybrid schemes.
- Prospects. We point out the open challenges of Wi-Fi-assisted indoor positioning, the multi-path effect, device deployment optimization, and data privacy. To these ends, we prospect promising directions in future work.

The rest of this paper is organized as follows: Section 2 describes the indoor positioning problem and its applications, and overviews the alternative methods. Section 3 classifies and analyzes Wi-Fi-assisted indoor positioning schemes, as well as review representative work. Section 4 points out the open challenges and forecasts the promising directions in future work. Section 5 concludes the whole paper.

## 2. Scenarios and General Advances

### 2.1. Application Scenarios

With the development of wireless technology, it is applied to various fields and plays an important role in people’s lives. Indoor positioning is one of the important applications of the wireless technology. It is defined as a process to obtain the position coordinates of people and objects in an indoor environment, e.g., hospitals, shopping malls, cinemas, flats, underground garages, and factories.

**Figure 1:**
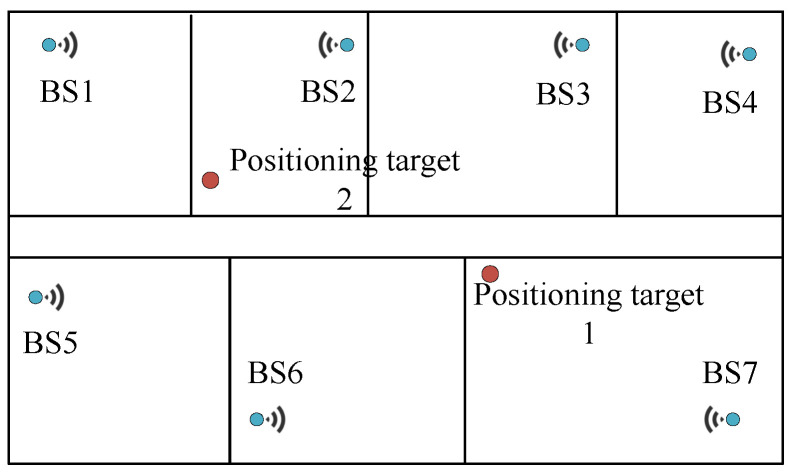
Indoor positioning [11].

A diagram of indoor positioning is illustrated in Figure 1. The base stations and positioning targets measure various parameters of the signal transmitted between them for positioning, e.g., angle and time-of-flight (ToF). Which parameter(s) to be measured, how to measure them, and how to utilize them vary over positioning schemes. In terms of the positioning principle, indoor positioning can be divided into angle-of-arrival (AoA) based [4], received signal strength indication (RSSI) based [8], and time-based schemes [9,10]. In terms of whether the positioning target carries the device for signal transmission, it can be divided into active and passive schemes.

Indoor positioning has a wide range of application scenarios. (i) In hospitals, indoor positioning can monitor the location of patients and medical staff in real-time. When patients experience an emergency, the hospital can quickly locate them and notice nearby medical staff to help them. (ii) In shopping malls, indoor positioning can aid customers in locating the store or purchasing products. (iii) In prisons, indoor positioning can help correctional officers track the whereabouts of prisoners in real time. (iv) In warehouses or factories, indoor positioning can assist in the management of valuable items and equipment, preventing them from being lost or stolen. (v) In underground garages, indoor positioning can help solve the problem of finding parking spaces, vehicle locations, and exits from the underground garage. (vi) In disaster relief, indoor positioning can help people escape the disaster area and assist in real-time rescue missions.

Location information may contain various sensitive information, such as users’ religious beliefs, lifestyle habits, and interpersonal relationships. The leakage of that information may expose users to unwanted advertisements and spam, may cause social reputation or economic losses to users, and may even subject them to extortion [12]. In addition, governments worldwide have established increasingly strict regulations to protect data privacy. Therefore, user privacy should be treated with caution by the designer of the positioning system. In Section 4, we elaborate on the challenge and promising research directions of Wi-Fi-assisted indoor positioning in data privacy.

### 2.2. Key Issues

In indoor positioning, the main objective is to achieve the accuracy of the LBS in a certain practical scenario. To this end, hardware cost, deployment difficulty, distance limitation, and device power consumption should also be taken into account. Anyway, the key issues of indoor positioning are mainly in two aspects, i.e., anti-interference and practical deployment.

#### 2.2.1. Anti-Interference

The key issue in anti-interference is to relieve the impact of the complex indoor environment on signal transmission. The direct path is defined as the straight transmitter-to-receiver path for signal propagation. Indoor obstacles may weaken the direct-path signal and meanwhile produce refraction and reflection. As to a superposition of the signals from these paths, it is difficult to determine the direct path, also known as the multi-path effect [4,13]. Other types of radio frequency (RF) signals may also make indoor positioning instability and inaccuracy.

#### 2.2.2. Practical Deployment

The key issues in the practical deployment of indoor positioning mainly lie in three aspects:

First, different buildings have various indoor structures and layouts. Thus, it is necessary to do an on-site survey when designing the deployment plan of the positioning system. The completeness of the on-site survey may affect the performance of the positioning system. However, the survey may incur an amount of effort and time costs. Second, the indoor environment may change frequently. The indoor positioning system needs to be able to adapt to changes in the environment. Alternatively, the system needs to be able to be redeployed at a low cost while maintaining its positioning performance. Third, the deployment of a positioning system needs to consider achieving a balance between cost and accuracy. High-accuracy positioning often has additional requirements for the device, increasing the device cost. In addition, in some scenarios (e.g., shopping malls), users may not prepare special devices for positioning. Excessive device requirements may make them unable to use the positioning system. Keeping low cost while providing high positioning accuracy is one research direction of this field.

### 2.3. Alternative Methods

In terms of radio access technology (RAT), we can classify the mainstream indoor positioning methods as non-Wi-Fi and Wi-Fi. The characteristics of these indoor positioning methods are described below.

#### 2.3.1. Non-Wi-Fi

Non-Wi-Fi methods include ZigBee [14,15], Bluetooth [16], ultra-wide band (UWB) [17], radio frequency identification (RFID) [18,19], ultrasonic [20], and infrared [21]. They differ in advantages of accuracy, power consumption, device cost, etc. Methods such as UWB [17], ultrasonic [20], and infrared [21] demonstrate high levels of precision. Regarding power consumption, Zigbee [14,15], RFID [18,19], Bluetooth [16], and UWB [17] methods exhibit low power consumption, with their power usage increasing sequentially. As for device cost, Zigbee [14,15], Bluetooth [16], and RFID [18,19] methods leverage cost-effective node devices or tag devices for localization.

Although these methods have various advantages, they also possess limitations, which are primarily related to two aspects: short effective distance and high device cost. Zigbee [14,15], Bluetooth [16], and RFID [18,19] methods suffer from limited effective range, whereas UWB [17], ultrasonic [20], and infrared [21] methods are associated with higher device costs. As a result of these drawbacks, these methods are unsuitable for establishing a universal indoor positioning system that is widely accessible and does not need specialized hardware support.

#### 2.3.2. Wi-Fi

Wi-Fi-assisted indoor positioning utilizes the signal transmission between the access point (AP) and the positioning target for positioning [22].

It has the following advantages. First, Wi-Fi-assisted indoor positioning has a longer effective distance than other alternatives, since the coverage of an AP indoors can reach up to 100 m away. Secondly, Wi-Fi is of lower device cost and more feasible for deployment. Third, the power consumption of Wi-Fi-assisted indoor positioning is relatively low, i.e., within 5 w for power and 100 mW for transmitting power for a general AP, which ensures the conservation of energy and barely interferes with other devices.

Note that there are also challenges of Wi-Fi-assisted indoor positioning, e.g., susceptibility to environments and low accuracy, which motivates extensive state-of-the-art work. In the rest of this paper, we concentrate on reviewing and analyzing Wi-Fi-assisted indoor positioning.

To sum up, advantages and disadvantages of these positioning methods assisted by various RATs are comprehensively summarized in Table 1.

## 3. Wi-Fi-Assisted Schemes on Different Principles

A typical Wi-Fi-assisted indoor positioning scheme is generally based on principles like AoA, RSSI, or time. Additionally, hybrid schemes combining multiple principles are also prevalent.

### 3.1. AoA

Wi-Fi-assisted schemes based on AoA were applied to indoor positioning for a long time. Many schemes with different focuses were proposed. In this part, we first describe the principle of AoA estimation and AoA-based indoor positioning. Then, we divide the representative work of AoA-based schemes into single-AP schemes and multi-AP schemes, and subdivide the multi-AP schemes based on various optimization directions of AoA estimation.

#### 3.1.1. Principle

The angle of signals received by the antenna array on the AP is referred to as AoA [4]. The indoor positioning based on AoA generally requires at least two APs. It uses the AoA of the signal and APs’ position to perform geometric positioning of the target. The signal’s AoA can be estimated by phase difference in the received signal from different antennas on the AP.

**Figure 2:**
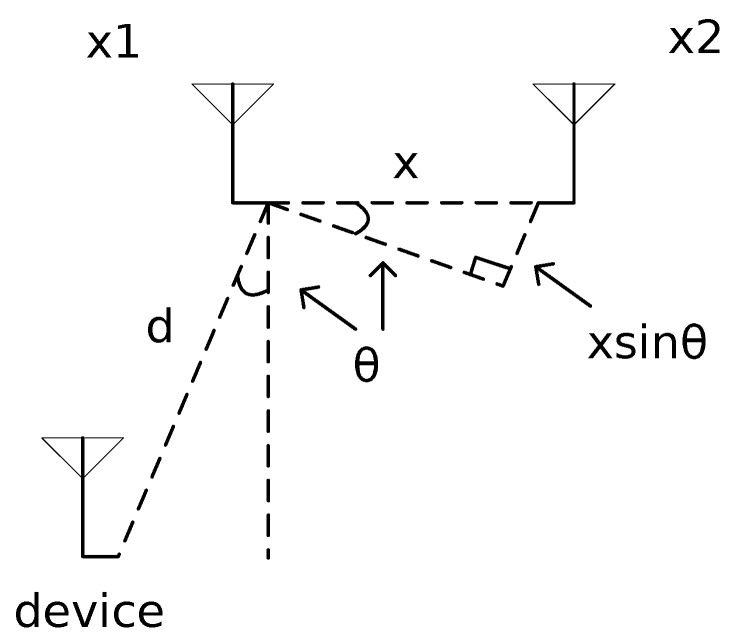
AoA estimation [4].

The principle of AoA estimation is illustrated in Figure 2. There is a device as a positioning target and a two-antenna AP in the figure. The AP has antenna $x_{1}$ and $x_{2}$. Let *d* denote the distance from $x_{1}$ to the device, λ denote the wavelength of the signal, *x* denote the distance between two antennas. It is obvious that xsinθ is the distance difference of signal propagation between two antennas. The distance difference can be calculated by the phase difference of received signals on the two antennas. Assuming the phase difference is denoted by α, then we have αλ/2π=xsinθ. We can express θ as

(1) θ=arcsin(αλ/2πx).

From Equation (1), we obtained the AoA of the signal. Specifically, when x=λ/2, θ=arcsin(α/π).

**Figure 3:**
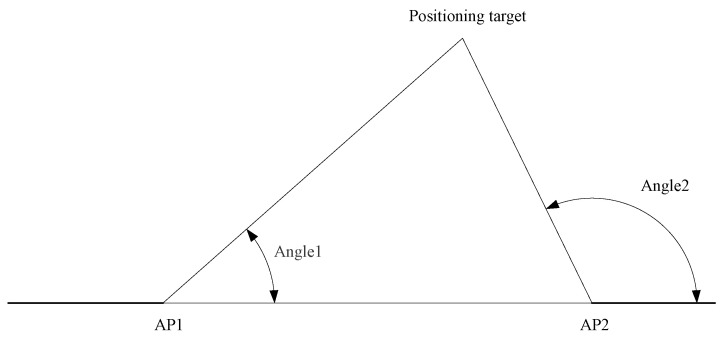
AoA-based indoor positioning [4].

The diagram of AoA-based indoor positioning is illustrated in Figure 3:

Its advantages include its suitability for short-distance positioning, along with a simple positioning principle and no need for time synchronization. However, it comes with several disadvantages, which can be categorized as follows. First, it has a high requirement on the estimation accuracy of the signal incident angle. Even a minor error in this estimation may cause a significant position estimation error. Therefore, as the distance between the positioning target and the AP increases, the decrease in positioning accuracy becomes apparent. Second, the positioning accuracy is limited by the size of the AP antenna array, so there might be a relatively high hardware cost to achieve a good positioning accuracy. Finally, due to the complex indoor environment, the multi-path effect may be severe, which may seriously harm the accuracy of AoA-based indoor positioning.

#### 3.1.2. Single-AP Schemes

The CUPID proposed by Sen et al., the spatial aliasing Wi-Fi localization (AWL) proposed by Chen et al., the TagFi proposed by Soltanaghaei et al. and the SAP-AoA proposed by Wang and Luan could use a single AP for localization [23,24,25,26].

The CUPID utilized the angle and distance of the direct path between the target and the AP for positioning. The angle was obtained by analyzing human mobility, while the distance was calculated by the energy of the direct path.

The AWL utilized the AoA of the signal from the positioning target to different antenna arrays on a single AP for positioning. It achieved decimeter-level positioning accuracy. The key to improving accuracy was using channel hopping to generate spatial aliasing and create virtual antennas.

The TagFi was a label positioning system that consisted of a single Wi-Fi device (e.g., laptop), a Wi-Fi transmitter, and labels. It applied a multiple signal classification (MUSIC) [27] based super-resolution algorithm to separate the label reflection from multi-path signals. On this basis, it estimated AoA and angle-of-departure (AoD) of the label reflection to determine the triangle with the Wi-Fi transmitter, the Wi-Fi device, and the label as vertices. Thereby, it obtained the label position.

The SAP-AoA located the target by using the distance between the two antennas on the AP and signals’ AoA received by them. They also explored the scheme of using FTM values in conjunction with SAP-AoA to achieve higher positioning accuracy.

#### 3.1.3. Multi-AP Schemes

The positioning accuracy of AoA-based schemes was improved generally by optimizing AoA estimation. There were three main optimization directions of AoA estimation in the schemes we surveyed.

Some schemes focused on improving angle estimation accuracy by dealing with the multi-path effect. Xiong et al. proposed Arraytrack [4]. It estimated the angle of the direct path for positioning. For the multi-path effect, it combined the spatial smoothing algorithm and the MUSIC algorithm to reduce its impact. Zhang et al. proposed iLocScan [28], which was the first to utilize the multi-path effect to assist in estimating the AoA and locating Wi-Fi devices. Yang and Gong proposed DeTrack [29], a real-time tracking system. It combined compressed sensing and expectation-maximization algorithms to mitigate the multi-path effect, achieving decimeter level accuracy.

Some schemes focused on improving angle estimation accuracy by extending the antenna array. Kumar et al. proposed Ubicarse [30]. It applied a new formulation of the synthetic aperture radar to emulate large antenna arrays on commercial mobile devices. With the help of emulated antenna arrays, the mobile device could obtain the direction of neighboring APs relative to the device for positioning. Gu et al. proposed TyrLoc [31]. It was an accurate multi-technology switching localization system that estimated the AoA of Wi-Fi, Bluetooth low energy (BLE), and long-range radio (LoRa) device signals for positioning. For improving the accuracy of the AoA estimation, it applied the RF switch to manage antenna switching, creating a large virtual antenna array on PlutoSDR.

Some schemes innovated AoA estimation methods. Karanam et al. achieved high-accuracy AoA estimation by measuring the amplitude of the received signal [32]. Tai et al. proposed unequal AoA tracking (UAT) [33]. It mathematically quantified the reliability of the AoA estimation on each AP and selected those reliable APs for localization. Tong et al. proposed MapFi [34]. It estimated AoA through CSI to obtain the position of APs and the angle of antenna arrays in the positioning system, reducing the labor cost of on-site surveys. Zhang et al. proposed localization framework WiCo [35]. The reliability of AoA estimations from different devices was actually unequal. WiCo utilized a normalized distribution confidence and full reference confidence to quantify this inequality, and resolved it by assigning varying weights to different APs through a re-weighting strategy.

The representative work of Wi-Fi-assisted schemes based on AoA is summarized in Table 2. If there are no special instructions, the accuracy is expressed by median error. It represents the threshold at which the linear distance between half of the estimated positions and the true position remains below.

### 3.2. RSSI

Wi-Fi-assisted schemes based on RSSI can be divided into fingerprint-based schemes and model-based schemes [8], among which fingerprint-based schemes are very popular. In this part, we describe their principles and state their representative works separately. We first introduce the early and latest work of fingerprint-based schemes and then describe special schemes including schemes mixed with other technologies, CSI-fingerprint-based schemes, and schemes on fingerprint positioning performance evaluation. Afterwards, we point out the two main optimization directions as well as their corresponding studies. Finally, we outline model-based schemes.

#### 3.2.1. Principle

Fingerprint-based indoor positioning [36] first collects fingerprints exploiting the correlation between RSSI and the physical location in the offline stage to construct a fingerprint database. Then, it compares the real-time measurement value with the signal strength data stored within the fingerprint database to estimate the location of the target in the online stage. It is noteworthy that, among all categories of positioning schemes, except fingerprint-based schemes, others are all based on ranging.

**Figure 4:**
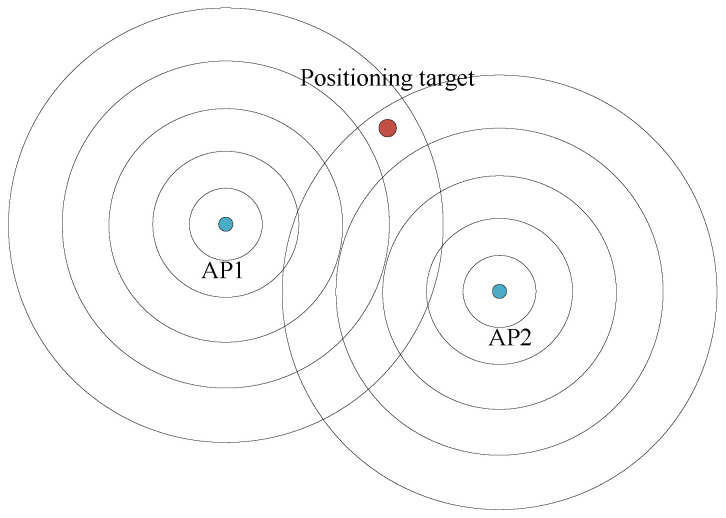
RSSI: fingerprint-based indoor positioning [36].

The diagram of fingerprint-based indoor positioning is illustrated in Figure 4:

Model-based indoor positioning [37] establishes a mathematical model that can predict the distance from the positioning target to the AP according to the signal strength. The distance is used to estimate the position of the positioning target by using trilateration or other methods. Those positioning schemes typically need at least three APs.

**Figure 5:**
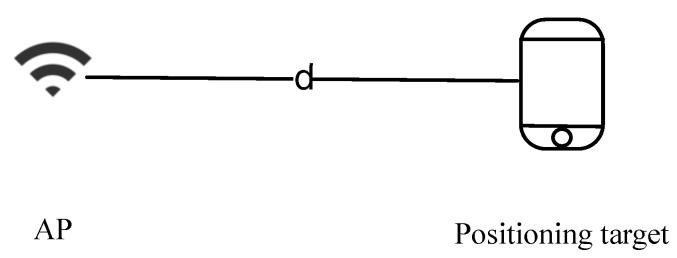
RSSI: model-based indoor positioning [38].

The diagram of model-based indoor positioning is illustrated in Figure 5. The logarithmic distance path loss (LDPL) model is expressed as

(2) $$PL\left(d\right)=PL\left(d_{0}\right)+10n\log\left(d/d_{0}\right)+X,$$

where PL(d) denotes the signal strength at distance *d* from the AP, $d_{0}$ denotes the reference distance, and *X* denotes a noise, e.g., the Gauss distribution. In addition to the LDPL model, there are also other models such as the free space path loss model and the linear distance path loss model [38].

The advantages of RSSI-based indoor positioning are no need for time synchronization, low hardware requirements, and no additional modification to the AP. In addition, fingerprint-based indoor positioning offers the advantage of theoretical immunity to the multi-path effect, while model-based indoor positioning has the advantages of simple principle, easy implementation, and deployment.

As for the disadvantages, fingerprint-based indoor positioning needs to collect fingerprints at multiple locations in advance, resulting in significant costs. This makes it difficult for fingerprint databases to adapt to environmental changes. Once there are significant environment changes, the fingerprint database may become outdated. It requires collecting fingerprints again. In addition, the movement of the positioning target may cause a Doppler frequency shift, which makes the feature-location relationship unstable. It may result in a significant decrease in the positioning accuracy of fingerprint-based indoor positioning.

The main drawback of model-based indoor positioning is low positioning accuracy. On one hand, the RSSI that can be obtained from commodity hardware is very coarse. On the other hand, the RSSI is greatly affected by the surrounding environment and the changes in the transmit power due to the device itself. The above two reasons result in inaccurate RSSI measurement, thereby reducing the positioning accuracy.

#### 3.2.2. Fingerprint-Based Schemes

Among fingerprint-based schemes, the earliest one was the RADAR [11] proposed by Bahl et al., which had meter-level accuracy. Afterwards, Youssef et al. proposed Horus [39], which was enhanced on the basis of RADAR. It utilized probability technology to estimate the position by a maximum likelihood-based method. Seifeldin et al. proposed a passive positioning system called Nuzzer [40], which consisted of APs and monitoring points (MPs). MPs monitored the strength of the signal sent by APs. Nuzzer constructed the fingerprint database in the offline stage and then used the algorithm based on Bayesian inference to estimate the most likely user location given the signal strength measured by MPs and the constructed fingerprint database.

After 2020, Chen et al. proposed a Wi-Fi passive positioning system FiDo [41]. Users differ in location fingerprints. The system eliminated this difference by locating many different users with labeled data from a few users. Shi et al. developed a precise non-causal positioning system [42]. It utilized building information to prevent illogical phenomena (e.g., sudden jumps) in position estimation. Yang et al. proposed a pyramid-structured fingerprint database fingerprinting pyramid map (FPM) [43]. Users could select fingerprint data with varying levels and densities for positioning, depending on their preferences for accuracy or efficiency in positioning. Tahat et al. investigated the impact of dual frequency information on the performance of fingerprint-based schemes based on different machine learning algorithms [44]. Zhao et al. proposed a lightweight Wi-Fi positioning privacy algorithm called the location preservation algorithm with plausible dummies (LPPD) [12]. It utilized generated reasonable virtual locations to protect the true location of users.

Some fingerprint-based indoor positioning schemes were mixed with other technologies. Yang et al. proposed a factor-maps based positioning system based on UWB and Wi-Fi [45]. Ranging information was provided by UWB, while fingerprint information was provided by Wi-Fi. Wu et al. designed an indoor positioning system consisting of Wi-Fi, geomagnetism, and pedestrian dead-reckoning (PDR) [46]. It first constructed the fingerprint database and the corner reference trajectory-geomagnetic database through PDR trajectories. Then, it applied a Kalman filter-based method to fuse this information for localization. Wu et al. proposed CWIWD-IPS [47], which applied a deep learning framework to fuse crowd-sensed inertial data and Wi-Fi fingerprint samples. Specifically, it first built inertial and Wi-Fi fingerprint databases, then exploited them to train the ResNet-based inertial neural network and the BiLSTM-based Wi-Fi fingerprint neural network. The results from two neural networks were fused by a Kalman filter for localization. Wang et al. proposed a hierarchical positioning scheme that integrated Wi-Fi, magnetic matching (MM), and PDR [48]. The scheme applied an adaptive extended Kalman filter to fuse PDR and positioning results from Wi-Fi and MM.

Some schemes used the CSI fingerprint rather than the RSSI fingerprint. Because the former contains richer and more robust wireless signal information, e.g., amplitude and phase responses of channels over different frequencies. Regani et al. proposed a room/zone-level positioning scheme based on Wi-Fi [49]. The scheme was device-free and calibration-free. It extracted features that indicated motion and breathing patterns from CSI to locate a person. Ayyalasomayajula et al. proposed DLoc [50], a wireless localization algorithm based on deep learning. It trained a neural network using ToF and AoA obtained from CSI. The neural network learned the relationship between signals and truth locations to build an environment model for positioning. Guo et al. put forward a federated TL framework FedPos for CSI fingerprint-based indoor positioning scheme [51]. It aggregated the non-classification layer parameters of models trained from different environments to build a versatile encoder. The encoder constructed personalized models for users, solving problems of privacy leakage and personalized training.

Some work analyzed the performance indicators of fingerprint-based indoor positioning. Krumm proposed pre-deployment and post-deployment models to estimate the accuracy of fingerprint-based schemes [52]. The pre-deployment model considered the possible impact of signal noise, signal quantization, spatial quantization, and calibration efforts on accuracy. The post-deployment model modeled the deployed positioning system to predict the accuracy of it. Mendoza Silva et al. described a method for performing a local-level analysis of the fingerprint-based scheme’s positioning errors [53]. This analysis investigated the accuracy of the positioning system at specific positions (e.g., corners) in the area it covered.

To improve the performance of fingerprint-based indoor positioning or reduce the cost of constructing and maintaining the positioning system, there are two main optimization directions for fingerprint-based indoor positioning schemes, namely fingerprint database construction and updating, and fingerprint matching algorithms.

#### 3.2.3. Fingerprint Database Constructing and Updating

For fingerprint database construction, Rizk et al. proposed LiPhi++ [54]. It greatly reduced data collection costs by utilizing the sensing capabilities of the transportable laser range scanner (LRS). Li et al. discussed a challenge in Wi-Fi fingerprint-based indoor positioning [55], which was how to sample a sufficient number of RSSI measurements in the offline stage. To this end, they proposed Kullback–Leibler divergence (KLD) to characterize the difference between the real distribution and the sampling distribution. Quezada-Gaibor et al. proposed a data cleaning algorithm based on the correlation among all samples in the fingerprint database [56]. The correlation among samples was calculated by the correlation between the RSSI fingerprint and AP’s identifier. Fingerprints with lower correlation would be removed to reduce the storage of the database.

Some schemes used the crowd-sourcing strategy for fingerprint database construction. Yang et al. proposed locating in fingerprint space (LiFS) [36]. In the offline stage, it utilized mobile phones carried by users to collect RSSI fingerprints and to record user movement path by measuring walking steps through an accelerometer. Thereby, it inferred the spatial relationship of RSSI fingerprints and constructed the fingerprint database. Rai et al. proposed Zee [8]. It collected fingerprints in the same way as LiFS. Specifically, it relied on indoor map and inertial sensors in the phone to infer the location and construct the fingerprint database during the offline stage.

Some schemes created virtual fingerprints to reduce database construction costs. The MonoFi proposed by Fahmy et al. first applied the k-nearest neighbor (KNN) regression model to generate virtual RSSI fingerprints at non-surveyed points in the space [57]. Then, it utilized the recurrent neural network (RNN) to learn user positions from temporal sequences of RSSI measurements generated by the fingerprint. Caso et al. proposed using the multi-wall multi-floor (MWMF) model in the empirical propagation model to generate virtual fingerprints during the offline stage [58]. Yong et al. proposed a new fingerprint database construction technology based on the synthetic minority over-sampling technique (SMOTE) [59]. It was applied to generate synthetic fingerprints in areas that were difficult to reach or were not regularly visited. Wei et al. proposed an effective fingerprint crowd-sourcing scheme [60]. In the offline stage, the system collected RSSI measurement data on the known path that users chose before. In the online stage, the system estimated the position based on Gaussian processes.

Some schemes used the clustering strategy to select fingerprints. Liu et al. proposed a weighted k-nearest neighbor (WKNN) positioning strategy using the k-means clustering fingerprint database [61]. The strategy reduced the impact of the RSSI fluctuation and maintained a balance between positioning accuracy and computational complexity. Sadhukhan et al. proposed a new weighted fusion-based efficient clustering strategy (WF-ECS) to fuse the similar fingerprint measured on the reference point (RP) belonging to the same cluster [62]. Ramires et al. proposed a clustering model called the strongest AP set (SAS) [63]. It utilized the concept that the strongest APs indicated the user’s region, clustering fingerprints based on the fixed size set of APs. The strongest AP set of a fingerprint refers to the set of a fixed number of APs with the strongest signal strength measured at the fingerprint’s location. In other words, APs in the set had stronger RSSI values than other APs at the position of the fingerprint.

For fingerprint database updating, Ren et al. proposed the ACOGAN model [64]. The model utilized a remeasured part of the fingerprints to update the whole fingerprint database. Tian et al. proposed a new unsupervised domain adaptation model TransLoc for Wi-Fi fingerprint updating [65]. It transferred location knowledge from the initial fingerprint database to the current unlabeled fingerprint for low-cost Wi-Fi fingerprints automatic updating.

#### 3.2.4. Fingerprint Matching Algorithms

In the direction of fingerprint matching algorithms, to address the environment change, Li et al. proposed the passive positioning system DAFI [66]. It developed a deep learning model for fingerprint matching by training the model with labeled CSI data from the original environment and unlabelled CSI data from the changed environment so that the positioning system could adapt to the changing environment. Song et al. applied deep domain adaptation (DDA) in transfer learning (TL) to the fingerprint matching algorithm model, enabling the model to continuously update by the changing RSSI data [67]. This strategy enhanced the model’s adaptability to the environment change.

Some schemes optimized fingerprint matching algorithms by selecting specific APs. Saccomano et al. proposed a deep learning-based indoor positioning scheme [68]. It first utilized signal strength to generate rankings of APs associated with a fingerprint, then exploited an RNN to learn the relationship between rankings and fingerprint locations for positioning. Zhou et al. proposed a positioning scheme which utilized AP contributions to positioning accuracy as the weight of the KNN fingerprint matching algorithm [69]. The AP contributions were calculated by signal distributions on every RP. Hou et al. proposed the fingerprint localization system FCLoc [70]. It applied a robustness principle to filter out the noise in RSSI samples and selected reliable APs for positioning according to the stability of online RSSI data. Yao et al. proposed an AP optimization integration model consisting of a Gaussian mixture model (GMM) region classifier and a random forest feature learner [71]. The model identified the best AP in the large-scale and complex environment to improve positioning accuracy.

#### 3.2.5. Model-Based Schemes

Due to the complexity of indoor signal propagation, it was challenging to apply model-based indoor positioning schemes indoors. The model-based active positioning system EZ proposed by Chintalapudi et al. was an early classic scheme [37]. It utilized the LDPL model to model the physical constraints of wireless propagation and used the genetic algorithm to solve them.

After 2020, Yang et al. proposed to fuse the internal state information of the system measured by the electronic compass and LDPL through an extended Kalman filtering algorithm [72]. They achieved the optimal pose estimation and path tracking of mobile robots. Hyder et al. proposed using the RSSI smoothing technique of weighted moving average and feedback filters to make RSSI measurements more accurate, which improved distance estimation accuracy of RSSI distance model [73]. Lin et al. proposed the ranging model GTBPD. It was constructed by training a back propagation neural network through the transformed RSSI. The ranging model was combined with the linear least-squares algorithm and the sequential quadratic programming (SQP) algorithm for location determination [74]. Choi proposed a scheme that combined Wi-Fi and sensors for ranging and localization [75]. The Wi-Fi ranging model was constructed by training a convolutional neural network (CNN) with CSI. Wang et al. proposed a cooperative positioning and mapping algorithm based on the max-product belief propagation and Kuhn–Munkres algorithm [76]. It solved the problem of mapping wireless devices to known three-dimensional (3D) installation points by utilizing the probabilistic graphical model and RSSI among devices.

The representative work of Wi-Fi-assisted schemes based on RSSI is summarized in Table 3 and Table 4.

### 3.3. Time

Time-based indoor positioning schemes can be further divided into time-of-arrival (ToA) based schemes [9] and time-different-of-arrival (TDoA) based schemes [10]. In addition, ToF ranging [3] is often employed for measuring the distance between the positioning target and the AP in ToA-based schemes. It is also utilized in some schemes to measure the distance between each antenna of the AP and the positioning target. Then, the measured distance is used in the method similar to the trilateration for positioning.

Precise time measurement needs high synchronization among devices and wide channel bandwidth, which Wi-Fi lacks. However, the introduction of the FTM protocol improves the accuracy of the time measurement. In this part, we introduce the principle of time measurement and time-based indoor positioning. Then, we review the representative work of time-based schemes. We first outline the early and latest work of time-based schemes, after that, we describe existing schemes using the FTM protocol.

#### 3.3.1. Principle

ToA-based indoor positioning requires at least three APs and strict time synchronization between the positioning target and each AP. It measures the arrival time of the received signal between different APs and the positioning target. The time is converted into distance, which is then employed into trilateration to calculate the target’s position.

**Figure 6:**
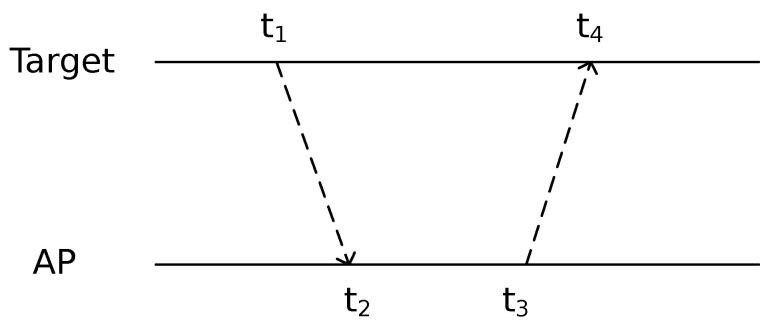
Time measurement [77].

The time measurement process is illustrated in Figure 6. Specifically, let *t* denote the arrival time of the signal between the positioning target and the AP. It is determined by four time points. They are $t_{1}$, $t_{2}$, $t_{3}$, and $t_{4}$ in chronological order. Under the premise of time synchronization between the AP and the positioning target, *t* can be obtained from the following expression:

(3) $$t=(t_{2}-t_{1}+t_{4}-t_{3})/2,$$

where $t_{1}$ and $t_{2}$ denote the time points that the positioning target sends the signal and the AP receives it, while $t_{3}$ and $t_{4}$ denote the time points that the AP sends the signal and the positioning target receives it. Through *t* obtained from Equation (3), the distance between the AP and the positioning target can be calculated.

The benefit of ToA-based indoor positioning lies in its simpler ranging process and easier development compared with TDoA-based indoor positioning. However, it has a drawback where the positioning target needs to achieve ranging with at least three APs in each positioning process. Each ranging requires multiple communications between the AP and the positioning target, which leads to higher energy consumption and greater vulnerability to interference. Additionally, the positioning target needs to handle signal transmission and reception, which causes higher hardware cost than TDoA-based indoor positioning.

**Figure 7:**
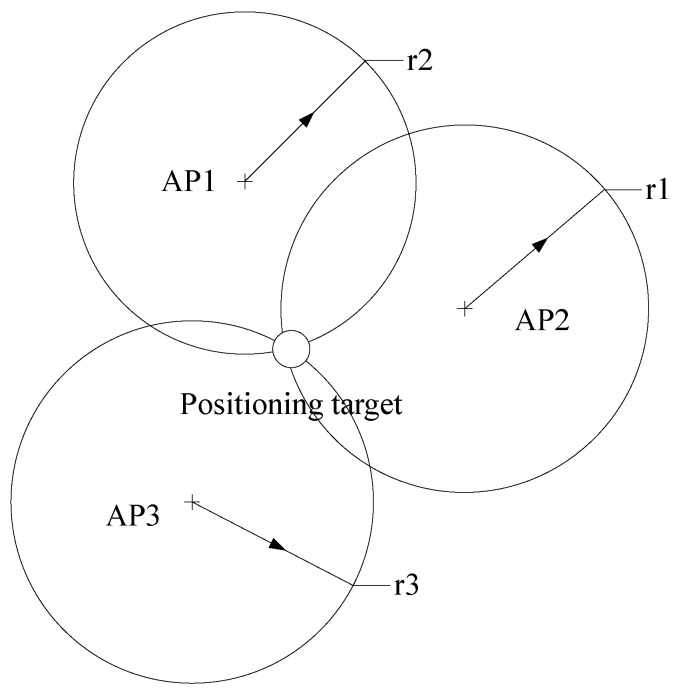
ToA-based indoor positioning [9].

The diagram of ToA-based indoor positioning is illustrated in Figure 7:

TDoA-based indoor positioning requires at least three APs. It first measures the time differences in the arrival of signals from positioning target to multiple APs for distance difference estimation between each AP and the target. Then, it uses the hyperbolic positioning method to locate.

The advantage of TDoA-based indoor positioning lies in its reduced time synchronization requirement compared to ToA-based indoor positioning. It only requires strict synchronization among APs. Furthermore, it can achieve positioning with just one signal transmission from the target, resulting in lower energy consumption compared to ToA-based indoor positioning. The disadvantage is that the algorithm is more complex than ToA-based indoor positioning.

**Figure 8:**
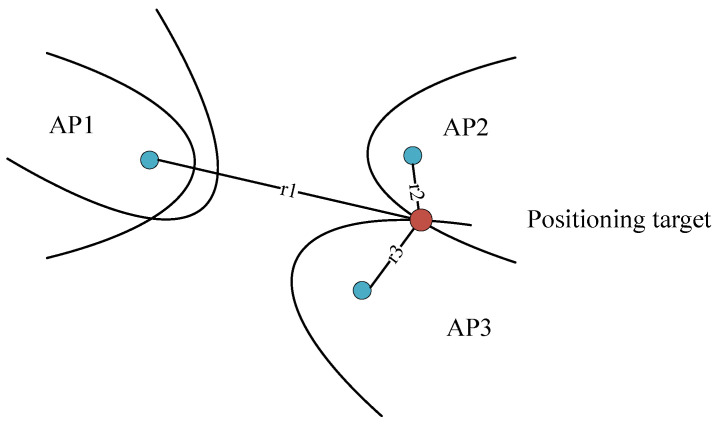
TDoA-based indoor positioning [10].

The diagram of TDoA-based indoor positioning is illustrated in Figure 8:

Finally, time-based indoor positioning suffers from the multi-path effect. In addition, they all have the drawback that minor time errors may lead to significant distance errors, given the fact that wireless signals travel at the speed of light. However, the narrow channel bandwidth of Wi-Fi is not enough to provide sufficient temporal resolution [10,78]. So it is hard to achieve high positioning accuracy. Fortunately, the Wi-Fi standard is continually evolving, introducing wider channel bandwidths. The latest Wi-Fi 7 has a 6 GHz frequency band, which supports 160 MHz channel bandwidth. It is expected to improve time measurement accuracy.

#### 3.3.2. Traditional Schemes

Xiong et al. proposed a TDoA-based indoor positioning scheme ToneTrack [10], which innovated in time measurement. By utilizing channel hopping in the continuous Wi-Fi frequency band, it combined the signal information from multiple frequency bands for more accurate time measurement. Vasisht et al. proposed Chronos based on ToF [78], which was the first to achieved decimeter level positioning with a single AP. Specifically, it used ToF to obtain the distance from each antenna on the AP to the positioning target for localization. To achieve accurate ToF estimation, it synchronized the AP and positioning target, letting them hop between the multiple frequency bands scattered around 2.4 GHz and 5 GHz. Rea et al. proposed TWINS [79], which was a ToA-based wireless indoor navigation system in industrial environments. It applied GMM to separate the direct path between the AP and the positioning target from the multi-path, measured the distance of the direct path through ToF, and estimated the position using the least squares method.

After 2020, Wang et al. proposed UbiTrack for locating single antenna internet-of-things (IoT) devices [80]. The UbiTrack system utilized ToF obtained from CSI to measure the distance between IoT devices. After that, the distance was leveraged by a new probability positioning algorithm based on the Bayesian estimation to determine the relative position of each device. Suraweera et al. developed a localization system that included multiple asynchronous sniffers [81]. Each sniffer listened to signals transmitted by the positioning target to measure the CSI and multi-path TDoA of it. This information was applied to two algorithms for localization. One algorithm employed a batch processing approach to jointly estimate the target path and the sniffers locations. Another algorithm employed particle filtering to track the target.

#### 3.3.3. FTM-Based Schemes

In 2016, the FTM protocol was introduced in Wi-Fi [6]. It enabled precise round-trip time (RTT) based ranging between the transmitter and receiver of Wi-Fi. Researchers proposed many Wi-Fi FTM-based indoor positioning schemes.

Cao et al. proposed a 3D indoor positioning algorithm for smartphones [82]. The distance measured by Wi-Fi FTM was first leveraged to the weighted centroid (WC) algorithm to estimate the rough two-dimensional (2D) position. Then, the result of WC was applied in the standard particle swarm optimization (SPSO) algorithm combined with density-based spatial clustering of applications with noise (DBSCAN) algorithm to obtain the accurate 3D position. Chan et al. proposed a scheme that used the neural network to predict the location of APs that support the FTM protocol [83]. It used a neural network trained with collected FTM data and a known AP position to identify non-line-of-sight (NLOS) paths of the signal and locate other APs. Ma et al. quantified the detailed Wi-Fi RTT ranging performance under various working modes and environments [9]. They proposed a new system bias elimination process to improve positioning accuracy. Si et al. proposed a weighted indoor positioning scheme based on Wi-Fi FTM suitable for NLOS environments [84]. It utilized compensation models to reduce ranging errors caused by clock drift and multi-path effect.

Some Wi-Fi FTM-based schemes were mixed with other technologies. Wang et al. implemented an indoor positioning scheme that integrated PDR and Wi-Fi FTM ranging using the extended Kalman filter framework [85]. Sun et al. proposed a new Wi-Fi FTM-based scheme assisted by geomagnetic positioning (GP) [86]. Chan et al. proposed an indoor positioning scheme that combined Wi-Fi FTM and PDR [87]. The Wi-Fi FTM data were exploited to estimate the location of the FTM responder infrastructure and train ranging models, while PDR inferred user positions and calibrated Wi-Fi FTM data.

The representative work of time-based indoor positioning is summarized in Table 5.

### 3.4. Hybrid Schemes

Hybrid indoor positioning schemes are based on a combination of AoA, time, or RSSI. They can be divided into passive schemes and active schemes. In addition, some special schemes use a single AP for positioning, use CSI to obtain AoA and time information, or combine Wi-Fi and other technologies. We outline these schemes separately. The representative work of hybrid schemes is summarized in Table 6.

#### 3.4.1. Passive Schemes

In passive schemes, Xie et al. successively proposed xD-Track and mD-Track [88,89]. The former fused information from different dimensions (e.g., ToF, AoA, AoD, Doppler frequency shift, signal attenuation) to locate the position of the human. The latter expanded the dimensions of information on the basis of the former. Li et al. proposed MaTrack [13], which utilized ToA to obtain AoA for positioning. Specifically, it applied a dynamic MUSIC algorithm to detect reflection signals from dynamic human bodies and utilized relative ToA to identify the shortest path of the reflection signal. The AoA of this path is regarded as the direction of the human target relative to the AP.

#### 3.4.2. Active Schemes

In active schemes, Pizarro et al. proposed UbiLocate [91]. It used Nelder–Mead-based search to estimate angles while constructing a fine-grained ToF ranging system with nanosecond resolution. In addition, it applied an AP selection mechanism to select APs with good estimation accuracy. Zhang et al. proposed NLoc based on ToF and AoA [92], whose key innovation was to convert multi-path reflections into virtual direct paths to enhance the localization performance. Choi et al. proposed an unsupervised learning framework that automatically optimized the ranging strategy [93]. Specifically, it selected the RSSI path loss model, FTM protocol, or neural network based on the actual situation to obtain the distance. Then, it calculated the location of the positioning target using the trilateration method. Yen et al. proposed a highly accurate 3D indoor positioning system [90]. The system estimated AoA of signals from Wi-Fi transmitters through RSSI to locate them.

#### 3.4.3. Special Schemes

Some schemes could perform localization with a single AP or device. Mariakakis et al. proposed single AP-based indoor localization (Sail) [94]. It combined ToF obtained from CSI with the RSSI to estimate the distance between AP and the positioning target. In addition, it utilized user movement to simulate the presence of multiple APs to perform single-AP localization. Li et al. proposed the single-device positioning system WiSight [95]. It applied a low-cost passive Wi-Fi antenna based on the frequency scanning antenna (FSA) technology, which used a single transceiver chain to measure AoA and ToF for positioning.

Some single-AP schemes utilized the multi-path effect to assist positioning. Chen et al. proposed $M^{3}$ [3]. It applied a super-resolution algorithm SAGE+ to jointly estimate channel parameters of the direct path and reflected paths of the signal for localization. Channel parameters included AoA, AoD, and ToF. Soltanaghaei et al. proposed the decimeter level Wi-Fi localization system MonoLoco [96]. The system applied a multi-path triangulation method, which utilized information from multi-path reflection to locate the device with a single receiver. The information from multi-path reflection was AoA and AoD of multi-path, as well as relative ToF between the direct path and each reflected path.

Some schemes got angle and time information from CSI. Kotaru et al. proposed SpotFi [97]. It used signals from the positioning target to multiple APs with three antennas, calculating their ToF and AoA to locate the target. A new 2D MUSIC algorithm was utilized to obtain ToF and AoA from the CSI. Zhang et al. proposed P2PLocate [98], a peer-to-peer localization system. The system enabled a single antenna device combined with a back-scatter tag to locate another single antenna device with decimeter accuracy. It leveraged CSI to estimate direction and distance of the target device. Jin et al. proposed an indoor positioning scheme that combined AoA obtained from CSI and FTM [99]. This scheme required no modification on the positioning target device. Wang et al. proposed a passive tracking scheme UKFWiTr based on unscented Kalman filter and CSI [100]. It estimated Doppler frequency shift and ToF through CSI and applied an unscented Kalman filter to optimize the AoA estimation. This information was exploited for localization.

Some schemes combined Wi-Fi with other technologies. Yu et al. proposed an accurate 3D indoor positioning and trajectory optimization framework [101], which combined Wi-Fi ranging and built-in sensors for localization. Wi-Fi ranging relied on RSSI and FTM. Choi et al. proposed a calibration-free positioning system using Wi-Fi ranging and PDR [102], where Wi-Fi ranging relied on RSSI or FTM. Each parameter in the system was optimized in real-time, ensuring robust system performance in various situations. Yu et al. proposed a hybrid positioning system that integrated Wi-Fi FTM, crowd-sourcing RSSI fingerprints, and micro-electro-mechanical-system (MEMS) sensors [103]. The system consisted of a Wi-Fi fingerprint database generation framework based on deep learning, a MEMS sensors-based localization method, and three different multi-source integration models. The integration model fused the information of light-weight pedestrian aimed inertial navigation system (PINS), Wi-Fi FTM, and RSSI fingerprints for localization.

## 4. Open Challenges and Promising Directions

Challenges and directions of Wi-Fi-assisted indoor positioning mainly lie in three aspects, namely, the multi-path effect, device deployment optimization, and data privacy.

### 4.1. Multi-Path Effect

In indoor active positioning, the perpetual challenge of addressing the impact of the multi-path effect on accuracy persists. The term “multi-path effect” refers to the phenomenon where a transmitted signal, after encountering reflection from objects, arrives at the receiver via different paths. The ToF and AoA of the signal on various reflected paths differ. For active positioning, determination of positioning target location depends on the ToF and AoA of the direct path. If the reflected path and the direct path cannot be accurately distinguished, positioning accuracy might be harmed. In comparison with the open outdoors, the indoor environment is complex, with various obstacles such as people, walls, tables, and chairs. They may cause a significant multi-path effect.

In active schemes, there are three main strategies to deal with the multi-path effect.

The first is to implement measures to mitigate the multi-path effect, e.g., TyrLoc [31], DeTrack [29] and AW-WFP [84]. TyrLoc employed the MUSIC algorithm, incorporating spatial smoothing and virtual antennas, to achieve a more precise estimation of the AoA. DeTrack utilized the expectation maximization method to improve the estimation accuracy of the AoA and ToF for the direct path. AW-WFP used compensation models to mitigate the impact of the multi-path effect. However, since they could not distinguish the direct path, they were not entirely immune to the impact of the multi-path effect.

The second is to eliminate the multi-path effect or employ schemes that remain insensitive to it, e.g., fingerprint-based schemes. The former involves identifying and exploiting the direct path to eliminate the multi-path effect, as demonstrated by CUPID, which harnessed the direct path for precise positioning [23]. Similarly, Chronos utilized the discrete Fourier transform to distinguish between the direct and reflected paths, effectively eliminating the multi-path effect [78]. TagFi eliminated the multi-path effect by utilizing back-scatter modulation and the spatial structure of signals to extract weak wireless reflections for positioning [25]. In fingerprint-based schemes, the multi-path effects only manifest when there is a change in the environment. The environment change leads to variation in the signal propagation path, resulting in inconsistent fingerprints at the same location before and after the change. Many schemes had been proposed to address the challenge posed by the environment change [64,65,66,67,68].

The third is to leverage the multi-path effect for positioning, e.g., $M^{3}$ [3] and NLoc [92]. The former scheme utilized AoA and AoD from multiple reflected paths, along with the ToF differences between these reflected paths and the direct path, for positioning. The latter scheme derived virtual direct paths by establishing a model that related the target location to the multi-path reflections.

In passive schemes, those based on AoA or ToF did not utilize the direct path for localization, because the direct path remained independent of the positioning target’s location. Instead, they utilized information such as the signal’s reflection path from the positioning target to determine its location. Their primary challenge within the context of the multi-path effect lies in distinguishing between the reflected signal from the target and the environmental obstacles, along with their respective propagation paths. The passive scheme based on ToA and AoA, MaTrack [13], applied a dynamic MUSIC algorithm for this purpose.

Although there are many strategies to deal with the multi-path effect, these strategies also have deficiencies that are worth studying and improving.

For the strategy of mitigating the multi-path effect, existing schemes are mature enough. They improved positioning accuracy by refining algorithms and optimizing devices for more accurate signal parameters estimation and measurement. The current algorithms mainly emphasize the enhancement of their accuracy. One potential research direction is to correct algorithmic outcomes through a specific method, e.g., a machine learning model. It could decrease the computational burden of the algorithm and lower device requirements by reducing the accuracy requirements of the algorithm.

For the strategy of eliminating the multi-path effect or employing schemes that remain insensitive to it, schemes aimed at eliminating the multi-path effect exhibited various limitations in their application scenarios. The CUPID used the movement of people to identify the direct path [23]. However, its accuracy suffered when people remained stationary. The algorithm of Chronos premised on the assumption that the direct path was strong enough, leading it to ignore those extremely weak paths in the signal propagation [78]. Therefore, when the direct path was too weak, its accuracy suffered. TagFi needed multiple antennas on both the Wi-Fi transmitter and receiver [25]. It had a smaller localization range because of its solution to the multi-path effect. Schemes with fewer limitations as well as broader application scenarios are still worth studying. For fingerprint-based indoor positioning, existing schemes addressed the environmental change from multiple perspectives. They included diminishing the role of RSSI in positioning, enabling the positioning system to automatically adapt to environmental changes, simplifying the process of updating fingerprints, etc. Recently, many schemes incorporated machine learning to address the environment change. However, there is currently no dedicated machine learning algorithm specifically designed for this issue. Existing solutions using the general algorithm encounter challenges of high complexity and high device performance requirements. Designing cost-effective and reasonable performance machine learning algorithms special for the environment change is a promising direction.

For the strategy of leveraging the multi-path effect for positioning, efforts should be focused on reducing the influence of positioning target movement on positioning accuracy. The information on the reflected path plays a pivotal role in localization. However, the movement of the positioning target may lead to the reflected path changing [3]. It reduces the accuracy of reflected path information estimation, resulting in a decrease in positioning accuracy. Ref. [92] discussed the influence of human movement on the accuracy. The decrease in accuracy during human walking is tolerable. However, as the speed of human movement increases, the error may increase accordingly. Therefore, it is meaningful to conduct research to mitigate the influence of movement on accuracy.

For passive schemes based on AoA or time, they leverage the movement of the positioning target to distinguish it from other stationary obstacles. So the positioning accuracy may suffer when the positioning target remains stationary or moves at a slow pace. It is worth studying to decrease the reliance of the positioning accuracy on the positioning target’s movement.

### 4.2. Device Deployment Optimization

The second challenge that needs to be addressed is fitting the indoor positioning scheme to Wi-Fi devices during the actual deployment phase. It mainly reflects in three aspects.

Firstly, indoor positioning schemes based on various principles have distinct fundamental requirements for the device, including the number of APs, antenna array size of the AP, and the sensor specifications for the positioning target device. During deployment, the number of APs should be adjusted according to the actual situation to achieve a balance between positioning performance and cost. Xiong et al. investigated the influence of APs’ number on positioning performance [10]. They advocated that the best solution was identifying the optimal group of APs, rather than randomly introducing more APs in the positioning system. The determination of the optimal group of APs was a research direction that they would explore in the future.

The performance of certain schemes is related to the AP antenna size and the sensor configuration of each AP. Antennas can be physical antennas or virtual antennas simulated through specific methods. For example, in AWL, a greater number of antennas on the AP yields more precise AoA estimations [24]. It could leverage channel hopping to generate virtual antennas to improve positioning accuracy. Some schemes require the presence of an inertial measurement unit (IMU) within the positioning target device, e.g., CWIWD-IPS [47] integrated personnel trajectories generated by PDR and Wi-Fi fingerprint information for positioning.

Secondly, there are various types of Wi-Fi devices. An actual deployed Wi-Fi system may include a range of brands of APs and positioning target devices. They may have various hardware and software configurations, e.g., their own set of standards for representing the parameters of the signal. Inconsistent standards may impair the estimation and measurement of parameters, further harming the accuracy. The current indoor positioning schemes lack research on running on devices with varying configurations. Constructing a universal positioning system that is able to seamlessly function across all devices remains a major challenge.

Finally, the deployed scheme may affect the inherent functionality of the Wi-Fi system. The Wi-Fi technology is mainly developed to provide internet services. The additional positioning requirements may trigger resource contention with the network services functionality of the Wi-Fi system, e.g., the channel hopping applied in work [10,24,78] and the MUSIC algorithm applied in work [4,13,31,97]. They hurt the performance of the network communication function.

At present, researchers proposed a series of indoor positioning schemes, each with distinct requirements in terms of the number of AP and the AP antenna array size. These requirements limit the application scenarios for those schemes. The most common home or small company office environments often exist only one AP with limited antenna array sizes. Consequently, there is an urgent need for schemes that perform well under single AP and limited antenna array size conditions to satisfy the positioning needs in these scenarios.

Researchers proposed several schemes to address this issue; however, these schemes possess certain limitations. AWL applied channel hopping to generate a large antenna to improve the accuracy of AoA estimations [24], while Chronos also relied on channel hopping to obtain accurate ToF estimations [78]. However, channel hopping is a time-consuming method that may hinder the data communication functions of Wi-Fi. Despite exploiting the multi-path effect, $M^{3}$ demonstrated a decline in positioning accuracy when tracking moving targets [3]. In addition, the simultaneous estimation of channel parameters across multiple dimensions requires the device with high performance. The MonoFi utilized the RNN to construct the fingerprint database and perform localization [57]. However, it was sensitive to the environment change. Designing a single-AP indoor positioning scheme that matches the performance configuration of mainstream APs remains an open issue. In addition, the scheme must ensure that it does not interfere with the standard communication function of Wi-Fi.

For the issue of diversity of Wi-Fi devices, there are two solutions. One is to construct an indoor positioning scheme that fully utilizes the consistency of each device. The other is to develop a unified positioning standard that all Wi-Fi devices adhere to expand consistency. For the issue of the impact of positioning systems on Wi-Fi communication functions, in addition to making efforts in constructing indoor positioning schemes, it is feasible to introduce positioning standards to the future Wi-Fi version. Furthermore, allocating dedicated resources for positioning services at the hardware and software levels of APs is also a good option.

In addition, there are currently not many devices supporting the FTM protocol and CSI data extraction. With the rapid advancement of Wi-Fi sensing technology and continuous increase in demand, it is believed that a growing number of Wi-Fi devices may support the FTM protocol and the CSI data extraction function in the future. They will also have excellent performance in positioning.

### 4.3. Data Privacy

The third challenge is the privacy issue of the positioning system. Ensuring the privacy of data is of great importance. At the personal level, the data from the positioning system may expose sensitive information about individual users, e.g., habit, health, and home addresses. At the public level, public security risks may arise from leakage of commercial secrets or sensitive information, like building structure information.

In addition, some indoor positioning schemes, especially those based on machine learning, require a large amount of data to support indoor positioning in various environments. However, there are two main issues in obtaining the data required for positioning. Firstly, to preserve data privacy and security, data from different sources should not be shared, e.g., data generated by users utilizing different applications or by users at distinct locations. Secondly, governments worldwide have implemented a range of regulations to enforce the protection of user data [104,105]. Those limitations related to data acquisition make it challenging for schemes that heavily rely on data support to gather enough data to construct positioning systems. In other words, it is difficult to obtain data without properly handling privacy issues. However, the processing of data to ensure privacy may inevitably harm the accuracy of indoor positioning schemes. Therefore, how to achieve accurate positioning while ensuring data privacy is a challenge.

Much effort has been dedicated to constructing solutions for privacy issues in indoor positioning. Some work employed cryptography-based schemes to address privacy issues. Li et al. were the first to propose encrypting the measured RSSI for privacy protection [106]. Similar work includes [107,108]. The latest work, the FedPos framework, also utilized the homomorphic encryption technology to address privacy issues [51]. However, the cryptography-based scheme incurs a large amount of computation and communication overheads. Some work utilized differential privacy-based schemes to address privacy issues [109,110]. Whereas it injected noise into the data, which might reduce the positioning accuracy. Recently, many works have introduced FL for privacy protection [51,110,111,112,113]. However, FL schemes suffer from the challenge of limited accuracy when applying the global model to different personalized scenarios. Some schemes attempted to solve this problem, yet the problem remains worthy of exploration.

Most research on privacy protection focused on fingerprint-based indoor positioning. However, privacy issues persist across schemes regardless of their principles [109]. Research on privacy issues about AoA or time-based indoor positioning still has great potential for expansion.

To sum up, addressing the privacy issues of Wi-Fi-assisted schemes remains a highly promising research direction. New schemes should find a balance among positioning accuracy, privacy protection cost, and privacy protection performance. This consideration can be approach from aspects such as data collection, data transmission, data encryption, and data access permissions. In addition, to the best of our knowledge, there is currently no unified benchmark for evaluating the cost and performance of privacy protection in Wi-Fi-assisted indoor positioning, which is also a promising research direction.

## 5. Conclusions

In this paper, we briefly described indoor positioning and its application scenarios. Then, we stated the key issues and alternative methods. We pointed out the advantages of Wi-Fi in indoor positioning, dividing Wi-Fi-assisted schemes into three categories. On this basis, we reviewed the authoritative work and the latest work of corresponding simple and hybrid schemes. Finally, we pointed out the open challenges as well as the promising directions of Wi-Fi-assisted indoor positioning in aspects of multi-path effect, device deployment optimization, and data privacy.

## Figures and Tables

**Table 1:** Advantages and disadvantages of positioning methods.

Positioning Methods	Advantages	Disadvantages
ZigBee [14,15]	Low power consumption, low cost for a single node.	Short signal transmission distance, signal susceptible to interference.
Bluetooth [16]	Low power consumption, small device size, low cost for single Bluetooth beacon.	Poor signal stability, short effective distance.
UWB [17]	High accuracy, interference resistance, low power consumption.	High device cost.
RFID [18,19]	Low power consumption, small size, and low cost of electronic tag.	High system complexity, hard to integrate electronic tag with mobile devices, short effective distance.
Ultrasonic [20]	High accuracy.	Signal susceptible to interference, high device cost.
Infrared [21]	High accuracy.	Signal susceptible to interference, high device cost.
Wi-Fi [22]	Long effective distance, low device cost, easy deployment, low power consumption.	Signal susceptible to interference, low accuracy.

**Table 2:** Representative work of Wi-Fi-assisted schemes based on AoA.

Positioning Schemes	Active/Passive	Device Requirements	Accuracy
CUPID [23]	Active	≥1 AP	5 m (1 AP)
AWL [24]	Active	1 AP	0.38 m (6 antennas)
TagFi [25]	Passive	≥1 AP, 1 Wi-Fi receiver	0.2 m
SAP-AoA [26]	Active	1 AP	0.85 m
Arraytrack [4]	Active	≥3 APs with 6 or 8 antennas	0.57 m (3 APs)
iLocScan [28]	Active	7 universal software radio peripheral (USRP) 2 units with 8 antennas	1.9 m (linear antenna array)
DeTrack [29]	Active	3 APs	0.9 m (80%)
Ubicarse [30]	Active	≥3 APs	0.39 m (3D device positioning)
TyrLoc [31]	Active	≥2 PlutoSDR with 8 antennas	0.63 m (Wi-Fi)
UAT [33]	Active	≥3 APs	1.3 m
MapFi [34]	Active	≥3APs	—
WiCo [35]	Active	3 APs	0.73 m

**Table 3:** Representative work of Wi-Fi-assisted schemes based on fingerprint.

Positioning Schemes	Active/Passive	Device Requirement	Accuracy
RADAR [11]	Active	3 base stations	1.3 m
Horus [39]	Active	Multiple APs	0.6 m
Nuzzer [40]	Passive	3 sending APs, 2 MPs	1.82 m
FiDo [41]	Passive	1 AP, 1 Wi-Fi receiver	Sub-meter level
Shi et al. [42]	Active	Multiple APs	0.7 m
FPM [43]	Active	Multiple APs	—
LPPD [12]	—	—	—
Yang et al. [45]	Active	Multiple APs and UWB anchors	1.8 m/0.9 m
Wu et al. [46]	Active	Multiple APs	3.34 m/4.5 m
CWIWD-IPS [47]	Active	—	4.06 m
Wang et al. [48]	Active	Multiple APs	1.02 m
Regani et al. [49]	Passive	1 AP, Multiple Wi-Fi receivers	—
DLoc [50]	Active	Multiple APs	0.8 m/0.94 m
FedPos [51]	Active	1 AP, 1PC, Multiple Raspberries	0.42 m
LiPhi++ [54]	Active	Multiple APs	0.67 m
Quezada-Gaibor et al. [56]	Active	Multiple APs	—
LiFS [36]	Active	Multiple APs	5.8 m
Zee [8]	Active	Multiple APs	3 m
MonoFi [57]	Active	1 AP	0.8 m
Caso et al. [58]	Active	Multiple APs	—
SMOTE [59]	Active	Multiple APs	—
Wei et al. [60]	Active	Multiple APs	—
Liu et al. [61]	Active	Multiple APs	1.45 m/8.54 m
WF-ECS [62]	Active	Multiple APs	—
SAS [63]	Active	Multiple APs	—
ACOGAN [64]	Active	Multiple APs	2.02 m (Field experiment)
TransLoc [65]	Active	Multiple APs	1.1 m (Office building)/4.0 m (Shopping mall)
DAFI [66]	Passive	1 AP, 1 Wi-Fi receiver	Sub-meter level
Song et al. [67]	Active	Multiple APs	2.65 m (11th month)
Saccomanno et al. [68]	Active	Multiple APs	—
Zhou et al. [69]	Active	Multiple APs	1.86 m
FCLoc [70]	Active	Multiple APs	<1 m
Yao et al. [71]	Active	Multiple APs	2.8–3.29 m

**Table 4:** Representative work of Wi-Fi assisted schemes based on model.

Positioning Schemes	Active/Passive	Device Requirement	Accuracy
EZ [37]	Active	≥4 APs	2 m/7 m
Yang et al. [72]	Active	≥3 APs	<0.05 m
Hyder et al. [73]	Active	3 APs	<0.5 m
GTBPD-LSQP [74]	Active	Multiple APs	2.099 m/2.112 m/2.635 m
Choi [75]	Active	Multiple APs	1.038 m
Wang et al. [76]	Active	Multiple anchors	–

**Table 5:** Representative work of Wi-Fi-assisted schemes based on time.

Positioning Schemes	Active/Passive	Device Requirement	Accuracy	Principle
ToneTrack [10]	Active	≥3 APs	0.9 m	TDoA
Chronos [78]	Active	1 AP with 3 antennas	0.65 m/0.98 m	ToF
TWINS [79]	Active	≥3 APs	1.8–3.8 m	ToA
UbiTrack [80]	Active	No AP	<2 m (RE = 0.5 m)	ToF
Suraweera et al. [81]	Active	Multiple sniffers	0.5 m/0.2 m	TDoA
DBSCAN-assisted SPSO [82]	Active	Multiple APs	1.147 m (2D)/0.305 m (altitude)	ToA/FTM
Chan et al. [83]	Active	Multiple APs	—	ToA/FTM
CbT & WCCG [9]	Active	≥4 APs	1.2 m (static)/1.3 m (dynamic)	ToA/FTM
AW-WFP [84]	Active	Multiple APs	1.31 m/3.72 m	ToA/FTM
Wang et al. [85]	Active	≥3 AP	1.5 m	ToA/FTM
EMEA-WLS [86]	Active	Multiple APs	1.82 m	ToA/FTM
Chan et al. [87]	Active	Multiple FTM receivers	0.75 m/0.77 m/0.94 m	FTM

**Table 6:** Representative work of hybrid indoor positioning schemes.

Positioning Schemes	Active/Passive	Device Requirement	Accuracy	Principle
xD-Track [88]	Passive	1 AP with 1 sending antenna and 1 AP with 4 receiving antennas	—	ToF/AoA
mD-Track [89]	Passive	A pair of transmitter and receiver using wireless open access research platform (WARP) with 8 antennas or AP with 3 antennas	0.36 m (WARP)/0.67 m (AP)	ToF/AoA
MaTrack [13]	Passive	1 signal transmitter and 2 APs with 3 receiving antennas	0.6 m	ToA/AoA
Yen et al. [90]	Passive	Wi-Fi transmitters and 3-antenna arrays	0.089 m/0.354 m	RSSI/AoA
UbiLocate [91]	Active	≥2 APs	0.75 m/1 m	ToF/AoA
NLoc [92]	Active	≥3 APs	—	ToF/AoA
Choi et al. [93]	Active	≥3 APs	2.397 m (RSSI)/1.547 m (FTM)	ToA/FTM/RSSI
Sail [94]	Active	1 AP with 3 antennas	2.3 m	ToF/RSSI
WiSight [95]	Active	Multiple localizing device with FSA	0.95 m	ToF/AoA
MonoLoco [96]	Active	1 AP	0.5 m	ToF/AoA
SpotFi [97]	Active	≥3 APs with 3 antennas	0.4 m	ToF/AoA
$M^{3}$ [3]	Active	1 AP with 3 antennas	0.71 m	ToF/AoA
P2PLocate [98]	Active	Back-scatter tag, a single-antenna device (receiver)	0.88 m	ToF/AoA
Jin et al. [99]	Active	2 APs with two external antennas	<0.5 m	FTM/AoA
UKFWiTr [100]	Passive	1 transmitter with 1 antenna and 1 receiver with 3 antennas	0.49 m	ToF/AoA
AUKF [101]	Active	Multiple APs	Meter level	ToA/FTM/RSSI
Choi et al. [102]	Active	Multiple APs	1.04 m	ToA/FTM/RSSI
H-WPS [103]	Active	≥4 APs	Meter level	ToA/FTM/RSSI

## Data Availability

No new data were created or analyzed in this study. Data sharing is not applicable to this article.
